# Development of the antimicrobial resistance burden score through a modified eDelphi

**DOI:** 10.1038/s44259-026-00184-w

**Published:** 2026-03-03

**Authors:** William J. Waldock, Mark Gilchrist, Frances Davies, Cesar de la Fuente-Nunez, Hutan Ashrafian, Ara Darzi, Bryony Dean Franklin

**Affiliations:** 1 Institute of Global Health Innovation, Imperial College London, London, UK; 2 Imperial College Healthcare NHS Trust, London, UK; 3 Department of Infectious Diseases, Imperial College London, London, UK; 4 Machine Biology Group, Departments of Psychiatry and Microbiology, Institute for Biomedical Informatics, Institute for Translational Medicine and Therapeutics, Perelman School of Medicine, University of Pennsylvania, Philadelphia, PA USA; 5 Departments of Bioengineering and Chemical and Biomolecular Engineering, School of Engineering and Applied Science, University of Pennsylvania, Philadelphia, PA USA; 6 Department of Chemistry, School of Arts and Sciences, University of Pennsylvania, Philadelphia, PA USA; 7 Penn Institute for Computational Science, University of Pennsylvania, Philadelphia, PA USA; 8 NIHR Northwest London Patient Safety Research Collaboration, London, UK; 9 UCL School of Pharmacy, London, UK

**Keywords:** Computational biology and bioinformatics, Health care, Medical research

## Abstract

Current antimicrobial resistance (AMR) surveillance relies on fragmented indicators that fail to capture institutional AMR burden complexity. To develop the AMR Burden Score, a three-round modified eDelphi study engaged interdisciplinary experts (Round 1: *n* = 17, Rounds 2 and 3: *n* = 7), including clinicians, microbiologists, pharmacists, health economists, and public health specialists. The AMR Burden Score comprises six weighted domains: Resistance (25%), Effectiveness (30%), Monitoring (30%), Adoption (5%), Processes (5%), and Systems (5%). Strong consensus emerged for core indicators, including incidence of resistant infections (unanimous Round 3 agreement, median 8.0), pathogen-specific resistance rates (median 7.0), and staff training programmes (median 8.0). The AMR Burden Score provides a structured framework for institutional AMR assessment, though implementation requires context-specific adaptation and further validation.

## Introduction

Antimicrobial resistance (AMR) is one of the most urgent and complex public health threats, with profound consequences not only for individual patient outcomes but also for the viability of healthcare systems globally. What was once considered a predominantly microbiological issue has evolved into a multifaceted global crisis, intersecting with agriculture, environmental health, economic development, and international security^1^. Therefore, key global actors, including the World Health Organisation (WHO), the United Nations General Assembly, and the World Bank^2^, have repeatedly called for coordinated national action plans that prioritise robust surveillance systems, antimicrobial stewardship (AMS), and infection prevention and control (IPC). A landmark global systematic analysis estimated that in 2019 alone, AMR was associated with 4.95 million deaths, including 1.27 million directly attributable to drug-resistant infections^3^. Compounding the challenge is the breadth of resistance: no longer confined to a few organisms, resistance is now widespread across common pathogens such as *Escherichia coli*, *Klebsiella pneumoniae*, *Staphylococcus aureus*, and *Streptococcus pneumoniae*^3,4^. Of particular concern is the rise of pan-resistant strains, those impervious to all known antibiotics, leaving clinicians with no effective therapeutic options^5^.

From an economic perspective, the burden of AMR is equally staggering. A 2016 UK government-commissioned report projected that, if unaddressed, AMR could cause up to $100 trillion USD in cumulative global GDP losses by 2050^6^. These costs stem not only from increased healthcare expenditures (e.g., prolonged hospitalisations, more expensive interventions) but also from productivity losses, diminished workforce participation, and the potential unravelling of medical procedures reliant on prophylactic antibiotics, such as surgery, chemotherapy, and organ transplantation. Lower- and middle-income countries (LMICs), already challenged by fragile healthcare infrastructures, are anticipated to bear a disproportionate share of this burden^1,6^. Nevertheless, despite growing awareness and an expanding evidence base, the tools available to measure, monitor, and respond to AMR remain fragmented and insufficient. Existing surveillance systems (including WHO’s Global Antimicrobial Resistance and Use Surveillance System (GLASS), and the European Antimicrobial Resistance Surveillance Network (EARS-Net)) have made important strides in tracking resistance prevalence and antimicrobial use^2,7,8^.

However, these systems often operate in silos, reporting isolated indicators that lack integration across institutions, geographies, or timeframes. For instance, an observed increase in *E. coli* resistance to third-generation cephalosporins may raise alarm, but without context, such as associated patient outcomes, AMS compliance, or bespoke prescribing behaviours, the significance remains ambiguous^9^. Moreover, standalone prescribing and safety indicators can be misleading. A facility reporting high antimicrobial usage may, paradoxically, be practicing rigorous stewardship and achieving strong clinical outcomes, while another with ostensibly low resistance rates may suffer from limited diagnostic capacity or underreporting^9,10^. Integration and cooperation with the UKHSA Fingertips resource is most important^11^ to deliver rigorous stewardship and achieve strong clinical outcomes.

This study aims to develop an AMR Burden Score, a comprehensive composite metric that captures the multifaceted nature of antimicrobial resistance across healthcare systems through expert consensus, addressing the critical gap in AMR surveillance by providing a standardised, actionable tool for cross-sectoral benchmarking, institutional evaluation, and strategic planning. We hope that this tool may serve as an additional AMR tool (alongside other AMR/AMS standards and resources already in operation) for healthcare institutions to perform retrospective self-assessment of their AMR landscape (resistance, management, and resource allocation) and to measure the effectiveness of interventions over time.

## Results

### Overview of the Delphi process

The Delphi study followed a structured three-round electronic process to reach consensus on the AMR Burden Score framework. No major deviations from the planned eDelphi process occurred. However, qualitative feedback from Rounds 1 and 2 highlighted concerns regarding the global applicability of indicators, particularly their relevance in LMICs. These contextual challenges prompted the research team to add targeted questions for LMIC feasibility, such as considering which metrics are reliably collected in different nations, in Round 2.

### Expert participation

A total of 36 international experts were invited, and 17 (47%) participated. Participants' demographics were as follows: 8 male, 9 female; 2 USA, 15 UK; Infectious disease specialists (*n* = 5), clinical microbiologists (*n* = 4), infection pharmacists (*n* = 3), public health professionals (*n* = 2), health economists (*n* = 2) and hospital administrators (*n* = 1).

### Round 1

Round 1 achieved consensus to include 9 indicators (≥ 75% agreement with ratings 7–9, IQR ≤ 2). The highest-rated indicators included incidence of resistant infections (median 8.0), incremental cost versus susceptible infections (median 8.0), attributable mortality cost (median 8.0), and staff training and education (median 8.0). Expert feedback identified methodological concerns regarding terminology on prevalence versus incidence, and applicability to LMICs, given the plausibility of collecting data in different nations.

### Round 2 and 3

Following structural refinement based on Round 1 feedback, seven experts evaluated the proposed REMAPS framework (Table 1). The framework demonstrated varying levels of consensus across domains, with the strongest agreement on WHO AWaRe usage patterns and year-on-year resistance trends.

**Table 1:** Round 2 REMAPS framework domain summary

Domain	Weight (%)	Top-rated indicator	Median (IQR)	Consensus to include^a^	Key concerns
Resistance (R)	25	Year-on-year resistance trends	8.0 (2.0)	Yes	Rate vs. proportion terminology
Effectiveness (E)	30	Incidence of resistant infections	7.0 (2.0)	Yes	Definition of clinical effectiveness
Monitoring (M)	30	WHO AWaRe usage patterns	8.0 (2.0)	Yes	Metric awareness variation
Adoption (A)	5	Audit and feedback	7.0 (2.0)	Yes	Implementation barriers
Processes (P)	5	Staff education	6.0 (2.0)	No	Overlap with other domains
Systems (S)	5	Attributable mortality cost	6.0 (2.0)	No	Economic data availability

*R* resistance, *E* effectiveness, *M* monitoring, *A* adoption; *P* processes, *S* systems, *WHO AWaRe* World Health Organisation Access, Watch, Reserve, *DDD* defined daily doses, *AMS* antimicrobial stewardship, *CDSS* clinical decision support systems, *IQR* interquartile range.

^a^Consensus defined as ≥75% agreement (ratings 7–9) with IQR ≤ 2.

The final round achieved a refined consensus (Table 2; Tables S1 and Table S2) on priority indicators and domain weightings. Strong consensus emerged for incidence of resistant infections (unanimous agreement, median 8.0, IQR 0.0), year-on-year resistance trends (median 8.0), and pathogen-specific resistance rates (median 7.0).

**Table 2:** Round 3 final consensus summary

Domain	Final weight (%)	Final consensus indicators	Median (IQR)	Consensus^a^
Resistance	25	Year-on-year trends	8.0 (1.0)	Yes
		Pathogen-specific rates	7.0 (2.0)	Yes
Effectiveness	30	Incidence of resistant infections	8.0 (0.0)	Yes
Monitoring	30	WHO AWaRe patterns	8.0 (2.0)	Yes
		DDD per 1,000 patient-days	7.0 (3.0)	No
Adoption	5	Clinical decision support systems	5.0 (1.0)	No
Processes	5	Staff training and education	7.0 (2.0)	Yes
Systems	5	AMS cost-effectiveness	7.0 (1.0)	Yes
		Attributable mortality cost	7.0 (1.0)	Yes

^a^Consensus defined as ≥75% agreement (ratings 7–9) with IQR ≤ 2.

The REMAPS AMR Burden Score, the consensus outcome of this expert eDelphi, uses integrated hospital electronic health record data, yielding scores out of 100 points with domain contributions reported in brackets in a bracketed subdomain framework similar to the Glasgow Coma Score (i.e., [*R*(x/25) + *E*(x/30) + M(x/30) + *A*(x/5) + *P*(x/5) + S(x/5)]). Lower scores indicate reduced antimicrobial resistance burden (Table 3). See Supplementary Table S3 for an illustrative step-by-step conversion of raw data (e.g., incidence rates, percentage compliance) into the domain score. An illustrative example of a final overall score is provided in Table 4.

**Table 3:** The AMR burden score

Domain	Weight	Raw hospital data sources	Key metrics extracted	Conversion mechanism	Final score calculation
Resistance (R)	25%	• Microbiology (organism identification, susceptibility) • Episodes (ward data, episode context)	• Overall MDR rates ( ≥ 3 antibiotic classes) • MRSA prevalence among *S. aureus* • ESBL rates in Enterobacteriaceae • ICU vs general ward resistance • UTI and CAP resistance (via ICD-10)	1. Extract organism susceptibility data 2. Calculate resistance to ≥3 classes 3. Link ward data for specialty stratification 4. Cross-reference ICD-10 codes with microbiology 5. Generate resistance rates per 1,000 patient-days	Higher resistance = Higher score (worse) Score range: 0–25 points
Effectiveness (E)	30%	• Pharmacy prescribing (prescription timestamps) • Pharmacy administration (administration records) • Episodes (length of stay, discharge status) • Emergency(readmissions)	• Resistant infection incidence per 1000 patient-days • Length of stay impact (resistant vs. susceptible) • 30-day mortality rates • Time to effective therapy (hours) • IV-to-oral conversion rates • 30-day infection relapse rates	1. Link resistance flags with episode data 2. Compare admission-to-discharge intervals 3. Track mortality within 30 days of resistant cultures 4. Measure specimen-to-antibiotic time 5. Assess IV-to-oral conversions 6. Identify readmissions within 30 days	Lower effectiveness = Higher score (worse) Score range: 0–30 points Target: ≤6 h to effective therapy
Monitoring (M)	30%	• pharmacy prescribing (drug names, doses, frequencies) • microbiology (culture results timing) • episodes (length of stay denominators)	• WHO AWaRe classification • DDD per 1000 patient-days • DASC • Guideline compliance rates • Culture-guided prescribing percentage • ASI	1. Map antibiotics to AWaRe categories 2. Calculate DDDs using doses/frequencies/durations 3. Analyse antibiotic breadth and duration 4. Compare regimens vs indication guidelines 5. Match prescription timing with culture results 6. Apply spectrum weights to prescribing duration	Better monitoring = Lower score (better) Score range: 0–30 points
Adoption (A)	5%	• System utilisation logs • Decision support notifications • Quality improvement databases • EHR workflow data	• CDSS implementation rates • Alert response rates • Audit and feedback completion • Intervention implementation tracking	1. Extract alert response rates from logs 2. Link prescription decisions with notifications 3. Quantify stewardship review completion 4. Track intervention implementation	Better adoption = Lower score (better) Score range: 0–5 points
Processes (P)	5%	• HR training systems • Pharmacy dispensing records • Clinical pathway data • Prescription modification logs	• Staff education completion rates • AMS interventions (IV-oral switches) • Dose optimisations • Duration adjustments • Guideline adherence monitoring	1. Extract training completion from HR systems 2. Link competency assessments to prescribers 3. Identify prescription modifications 4. Analyse administration record changes 5. Track protocol compliance through pathways	Better processes = Lower score (better) Score range: 0–5 points
Systems (S)	5%	• Financial/cost data• Infection control data • Resource utilisation metrics • Diagnostic turnaround times • Pharmacy expenditure records	• Cost-effectiveness analysis • Attributable mortality costs • Rapid diagnostics savings • Productivity loss from extended stays • Therapeutic agent costs • Bed closure and transmission costs	1. Link financial data with resistance patterns 2. Calculate incremental costs per resistant infection 3. Combine mortality statistics with resource use 4. Measure diagnostic improvement savings 5. Quantify extended hospitalisation costs 6. Integrate infection control with resource metrics	Higher burden = Higher score (worse) Score range: 0–5 points

*MDR* multi-drug resistant, *MRSA* *Methicillin Resistant Staphylococcus Aureus*, *ESBL* *Extended spectrum beta-lactamase*, *ICU* intensive care unit, *UTI* urinary tract infection, *CAP* community-acquired pneumonia, *AWaRe* access/watch/reserve, *DDD* defined daily dose, *DASC* days of antibiotic spectrum coverage, *ASI* antimicrobial spectrum index.

‘Resistant infection’ is defined as an infection caused by a pathogen demonstrating in vitro non-susceptibility to at least one first-line or critical antimicrobial agent relevant to the infection site/syndrome, as determined by local/national guidelines.

**Table 4:** Illustrative case study: monitoring improving AMR burden at ‘metropolitan general hospital’ following an intervention; the lower the score, the better

Quarter	Total score	R	E	M	A	P	S	Illustrative changes to resistance metrics
Q1 2023 (Baseline)	72/100	22/25	25/30	20/30	2/5	2/5	1/5	e.g., 18% MDR rate, 8% MRSA, 3.2-day excess LOS, 15% 30-day mortality, 35% culture-guided prescribing
Q3 2024 (Post-Intervention)	51/100	15/25	18/30	13/30	2/5	2/5	1/5	e.g., 12% MDR rate, 5% MRSA, 1.8-day excess LOS, 8% 30-day mortality, 58% culture-guided prescribing

## Discussion

This study presents the development of the AMR Burden Score, a multidimensional composite index designed to quantify the burden of antimicrobial resistance (AMR) across institutional, regional, and national levels. This is intended to serve as an additional AMR tool (alongside other AMR/AMS standards and resources already in operation) for healthcare institutions to perform retrospective self-assessment of their AMR landscape (resistance, management, and resource allocation) and to measure the effectiveness of interventions over time. Traditional AMR surveillance methods typically rely on discrete, siloed indicators, such as resistance prevalence, antibiotic consumption rates, or stewardship compliance^11–13^. While each of these offers valuable insights, none alone captures the full complexity of AMR as a systemic threat. The AMR Burden Score addresses this gap by integrating a wide array of data sources, including clinical outcomes, microbiological trends, stewardship practices, and economic impacts, into a single, interpretable composite score^14^. In doing so, it provides a robust, dynamic model capable of supporting near real-time assessments and longitudinal monitoring^15^. However, the development process revealed fundamental tensions regarding framework coherence. Expert feedback highlighted a critical debate about whether AMR burden measurement should be integrated with stewardship process evaluation. While composite measurement offers a comprehensive assessment, there is potential for misrepresentation by conflation of distinct analytical objectives that serve different decision-making needs. Initial framework acceptance was strong, with 14 of 17 experts (82%) in Round 1 agreeing that metrics within each domain reflected relevant and measurable aspects of AMR and antimicrobial stewardship (AMS). However, significant methodological concerns emerged through qualitative feedback analysis regarding the combination of resistance epidemiology with management processes in a single composite score.

The score’s development was underpinned by the REMAPS framework following a scoping review by the authors, in consultation with other work^14–16^, which articulated six core domains: Resistance, Effectiveness, Monitoring, Adoption, Processes, and Systems. Through a structured, three-round modified eDelphi process, domain relevance, indicator selection, and relative weighting were refined in response to expert feedback, as illustrated in similar literature describing stewardship implementation programmes^17^. The consensus evolution revealed notable patterns across the three rounds (Round 1: *n* = 17; Round 2: *n* = 7; Round 3: *n* = 5). Most significantly, the incidence of resistant infections achieved a unanimous consensus in Round 3 (median 8.0, IQR 0.0), establishing this as the core AMR burden indicator. Other indicators achieving strong final consensus included year-on-year resistance trends (median 8.0, IQR 1.0) and staff training and education (median 7.0, IQR 2.0). The framework was reinforced by mortality-based calibration in the Effectiveness domain, derived from studies comparing outcomes in resistant versus susceptible infections^18,19^. Together, these components ensure that the score is both methodologically sound and clinically meaningful. The expert endorsement process revealed significant methodological challenges. The substantial attrition rate of 59% from Round 1 to Round 3 (from *n* = 17 to *n* = 5) created potential selection bias toward more engaged, implementation-focused experts. Critically, all health economists departed after Round 1, eliminating crucial economic evaluation perspectives from subsequent framework refinement. This professional background bias significantly influenced outcomes, with specialty backgrounds affecting indicator priorities throughout the consensus process^17^. The small final sample size raises questions about the robustness of late-round consensus findings and suggests the 75% agreement threshold may have been too permissive for meaningful consensus development. The expertise in Rounds 2 and 3 remained focused on the core clinical fields (Infectious Diseases/Microbiology/Pharmacy), ensuring the core clinical perspective was maintained despite attrition. The score was designed for interoperability with existing AMR surveillance systems^11^, housing routine data on resistance patterns, antimicrobial prescribing, and infection prevention measures^20–22^. Implementation feasibility varies by setting, with sophisticated infrastructure indicators receiving lower consensus ratings, while core prescribing practice indicators showed sustained expert support. The score aligns with national and global AMR strategies, including WHO’s Global Action Plan and national AMR action plans, operationalising strategic goals and coordinating multisectoral responses following One Health principles^23–25^.

The eDelphi methodology provided structured expert consensus mechanisms, engaging clinicians, microbiologists, pharmacists, economists, and public health professionals^17^. Pharmacists offered expertise in antimicrobial stewardship as well as including pharmacokinetic, dynamic and economic issues. However, purposive sampling may have over-represented certain perspectives, with noted gaps in veterinary AMR expertise and limited global representation. Expert feedback revealed crucial terminological concerns, particularly the need to distinguish prevalence from incidence rates and clarify microbiological versus clinical burden concepts. The Effectiveness domain achieved the strongest consensus, with resistant infections consistently rated as the primary burden indicator across all rounds. The WHO AWaRe (Access, Watch, Reserve) usage patterns showed strengthened consensus from Round 1 (median 7.0, IQR 2.0) to Round 3 (median 8.0, IQR 2.0), while more complex analytical indicators such as the Antimicrobial Spectrum Index lost support over successive rounds^26–28^. The model’s contextual flexibility was designed to accommodate varying health system contexts through modular implementation, particularly addressing resource limitations in low- and middle-income countries^10,29,30^. We acknowledge the omission of *Vancomycin-resistant Enterococci* (VRE) and the broad grouping of *Carbapenem-resistant Enterobacteriaceae (*CRE); the initial scoping was designed to capture broad categories that represent a significant burden across multiple countries. There is a trade-off between breadth and specificity, and local adoption should allow for substitution of locally critical pathogens (e.g., adding VRE or replacing a broad group with CRE).

Despite its strengths, this study has important limitations that warrant attention. The 59% overall attrition rate between Round 1 and Round 3 created potential selection bias, though the specific professional and geographic characteristics of participants who withdrew versus those who remained require further analysis to assess the nature and extent of this bias. The loss of all health economists after Round 1 eliminated crucial economic evaluation perspectives from framework refinement, potentially explaining the relatively weak consensus around economic indicators in later rounds. Development in low- and middle-income countries and additional non-UK settings is necessary to confirm the model’s global applicability^10,31^. Professional interest bias significantly influenced outcomes, particularly as only experts from the UK and the US participated, with specialty backgrounds affecting indicator priorities throughout the process. This raises questions about the objectivity of consensus-based approaches and suggests the need for additional development methods beyond expert opinion.

Second, data heterogeneity poses challenges, particularly in domains reliant on economic indicators, which may be inconsistently defined or collected across institutions^32,33^. The weighting system, although derived through expert consensus, remains partially subjective. Incorporating machine learning approaches in future iterations could provide a more data-driven method for refining domain influence^34^. The score’s temporal rigidity by relying on periodic data collection, limits responsiveness in acute settings or during rapid AMR shifts^35^. The AMR Burden Score needs statistical consideration of confounding factors (hospital size, resource allocation, and baseline incidence) when comparing scores between institutions, particularly in relation to the epidemiology and management domains. This requires a statistician’s input for future validation studies. The framework’s coherence concerns, raised consistently by experts, suggest that combining resistance burden measurement with stewardship process evaluation in a single composite score may conflate distinct analytical objectives. Future iterations should consider whether separate indices for epidemiological burden and management effectiveness would provide clearer insights for different decision-making contexts^36–38^. There is potential for a future ‘Alerting Function’ built upon the baseline AMR Burden Score. While the current retrospective score is not designed for real-time outbreak detection, the Management (M) and Policy (P) domains capture the infrastructure required for rapid response, which the score helps to benchmark.

Expanding implementation beyond the UK presents significant and multifaceted challenges, particularly for LMICs where surveillance infrastructure remains fragmented and under-resourced. The score is designed to be calculated annually or semi-annually by an Antimicrobial Stewardship (AMS) team, allowing for standardised comparison within an institution over time and between comparable institutions (e.g., those with similar resources and specialties) to identify areas for strategic health innovation and intervention. These settings require comprehensive framework modifications, including simplified indicators that align with limited laboratory capacity, reduced data collection requirements, and adaptation to diverse healthcare delivery systems^39^. The challenges are compounded by the scattered nature of AMR surveillance in LMICs, where weak laboratory capacity, poor health systems governance, inadequate health information systems, and limited financial resources create substantial barriers to effective monitoring programmes. For resource-limited settings (RLS), a simplified, reduced-metric score focusing on outcomes and education is a necessary future step. Future iterations should prioritise automation and interoperability through electronic data integration using globally recognised standards, particularly those promoted by WHO’s Global Antimicrobial Resistance and Use Surveillance System (GLASS)^40^. This approach would enable more effective linkage with electronic health records, laboratory information management systems, and existing surveillance networks, creating a unified data ecosystem that supports real-time monitoring. The integration should leverage automated reporting mechanisms already implemented in many clinical diagnostic laboratories, which have demonstrated clear advantages in speed, accuracy, and reduced reporting burden on healthcare facilities.

Embedding predictive analytics capabilities, including advanced time-series forecasting, machine learning algorithms, and pattern recognition systems, could transform the score from a retrospective assessment tool into a forward-looking decision support system^41^. These enhancements would enable early detection of emerging resistance patterns, prediction of resistance trends, and identification of intervention opportunities before critical thresholds are reached. The integration of genomic surveillance data and whole genome sequencing results could further enhance the score’s predictive capacity and support outbreak investigation efforts. Sustained stakeholder engagement remains critical for successful implementation, with organisational leadership preferences currently split between patient safety and medical director models (35% each). Internationally, antimicrobial stewardship typically aligns with patient safety frameworks; however, significant differences in understanding and operational interpretation influence programme effectiveness and delivery outcomes. The moderate framework acceptance rate (82% strong agreement) indicates that additional targeted engagement strategies, capacity building initiatives, and demonstration of clear value propositions may be necessary for achieving widespread adoption^25^.

The Systems domain offers substantial opportunities for enrichment through more sophisticated economic modelling approaches, including comprehensive cost-effectiveness analyses, budget impact evaluations, and return-on-investment assessments^41,42^. These enhancements would provide decision-makers with critical financial data to support programme justification, resource allocation, and sustainability planning. Integration with broader health economic frameworks would enable the AMR Burden Score to demonstrate its contribution to overall health system performance and patient safety objectives, thereby strengthening the case for continued investment and expansion of surveillance capabilities. The development should also incorporate lessons learned from existing surveillance evaluation tools and frameworks, ensuring that the scoring system addresses both technical performance metrics and collaborative effectiveness across multiple sectors and stakeholders involved in AMR prevention and control. The potential integration of the AMR Burden Score into national surveillance platforms offers a transformative opportunity to support and strengthen antimicrobial resistance monitoring capabilities. This multidimensional scoring framework provides comprehensive AMR burden evaluation across clinical, operational, and economic axes, enabling stakeholders to track temporal trends, conduct geographic comparisons, and identify resistance hotspots^20,24,34^. The score addresses a critical gap identified in recent global burden studies, which highlight the urgent need for standardised, comparable metrics to guide evidence-based policy decisions in combating AMR at national and international levels^43,44^. We aspire to the prospective in-use validation of the score within a selection of UK NHS Trusts; the reliability and standardisation of underpinning data sources for future score implementation are essential.

The AMR Burden Score represents a further tool in the measurement and monitoring of antimicrobial resistance. Developed through a rigorous process of expert consensus and empirical calibration, it provides a structured, integrative, and scalable framework for quantifying AMR burden across diverse healthcare systems. By consolidating clinical, microbiological, stewardship, and economic data into a unified metric, the score enables more standardised institutional reporting, facilitates tracking of stewardship interventions, supports evidence-based research prioritisation, and promotes cross-system benchmarking. Crucially, it transforms fragmented AMR data into actionable intelligence, equipping healthcare providers, policymakers, and public health agencies with a tool to respond more strategically to the evolving global threat of antimicrobial resistance. The immediate next steps are focused on piloting operational validation with a UK healthcare organisation and collaborating with national partners within the AMR space.

## Methods

### Delphi design and integration of expertise

This study, over 3 months, employed a three-round modified electronic Delphi (eDelphi) method to develop, validate, and calibrate the AMR Burden Score, a composite index designed to quantify the burden of antimicrobial resistance (AMR) across healthcare settings; the modification arose out of restrictions on resources. Imperial College London university ethics approval was granted in full (7819350). The methodological process followed best practices for Delphi studies as outlined by the DELPHISTAR^45^ guidelines. Participants included professionals in clinical microbiology, infectious diseases, pharmacy, health economics, epidemiology, infection prevention and control, and clinical informatics. These disciplines were selected to ensure comprehensive representation of theoretical, practical, and systems-level perspectives relevant to AMR.

If expertise in specific subdomains of AMR (e.g., veterinary AMR, global south representation) could not be integrated due to recruitment limitations, this was communicated to the panel during the Delphi process. Experts were defined as individuals with a minimum of five years of active engagement in AMR-related fields, with demonstrable involvement in policy, surveillance, clinical practice, or research pertaining to AMR.

### Delphi variant and rationale

A modified three-round electronic eDelphi approach was used, consistent with adaptations commonly employed in digital consensus-building methodologies. It was modified in that a scoping review, not a full systematic review, was used to develop the first set of statements. The electronic format enabled asynchronous participation from international experts. A maximum of three rounds was pre-specified to prevent participant fatigue. No additional rounds were planned unless response attrition or consensus thresholds necessitated adjustment.

### Expert sampling and recruitment

All experts meeting the above definition were invited to participate in Round 1. Only those who completed a given round were invited to participate in subsequent rounds. Experts were identified via purposive sampling based on their publication records (searched via PubMed), roles in national and international AMR initiatives, and participation in relevant policy or clinical committees. Experts were invited via personalised email containing information about the study’s aims, anonymity protocols, and expected time commitment. Participation was voluntary, and informed consent was implied by completion of the survey. No incentives were provided. Three follow-up reminders were sent to maximise retention across rounds.

### Questionnaire development and structure

This Delphi study employed a systematic three-round approach to develop and validate the REMAPS framework for antimicrobial resistance burden measurement. Round 1 evaluated indicators across six domains (Resistance, Effectiveness, Monitoring, Adoption, Processes, Systems) using nine-point Likert scales. The domains were derived from established pillars of national AMR action plans (Surveillance, Stewardship, IPC, Research, Policy) but were modified and re-framed to focus on quantifiable institutional metrics for self-assessment. Experts were given proposed preliminary weights based on literature review findings, allocating thirty percent each to Effectiveness and Monitoring, twenty-five percent to Resistance, and five percent each to the remaining domains. These represented working allocations for testing rather than validated weightings. Round 2 introduced explicit domain weighting evaluation to assess both individual indicators and overall domain allocations while providing feedback on framework coherence. Round 3 continued this development process, requiring them to evaluate proposed weights using agreement scales, suggest alternative percentage distributions, and rank domains by importance for antimicrobial resistance burden measurement. This progressive refinement evolved from researcher-proposed allocations to expert-validated consensus weights.

### Delphi rounds and objectives

A total of three Delphi rounds were conducted to evaluate the relevance of candidate domains and indicators, and to seek to establish consensus on the final framework. The maximum number of rounds was pre-specified as three, and continuation beyond that point was not planned. The third round was contingent upon retention levels and the degree of unresolved dissent. Quantitative data were analysed descriptively using medians, IQRs, and frequency distributions. Free-text responses were thematically analysed using an inductive coding strategy. Consensus for inclusion of an item in the score was defined a priori as ≥75% of respondents giving ratings 7–9 on the Likert scale with IQR ≤ 2 for indicator inclusion or acceptance; consensus on a lower score on the Likert scale would result in exclusion from the score. No group-specific weighting was applied during indicator rating.

### Ethics approval

Imperial College London's Research Governance and Integrity Team (RGIT) provided full research ethics approval (7819350).

## Supplementary information

Supplementary File

## Data Availability

All are reported in the manuscript.
