# Supplementary material for: Development of the antimicrobial resistance burden score through a modified eDelphi
Source: NPJ Antimicrob Resist. 2026 Mar 3;4:15. doi: 10.1038/s44259-026-00184-w (PMC12957418; doi:10.1038/s44259-026-00184-w)
Supplement: Supplementary file 1 — Supplementary File [file 44259_2026_184_MOESM1_ESM.docx]

**Supplementary Results**

### **Supplementary Table S1: Round 1 High-Priority Indicators by Domain (n=17)**

**Abbreviations:** AMR = Antimicrobial Resistance; AMS = Antimicrobial Stewardship; WHO AWaRe = World Health Organization Access, Watch, Reserve classification; CDSS = Clinical Decision Support Systems; MDR = Multi-Drug Resistant

| **Domain** | **Indicator** | **Median (IQR)** | **% Rating 7-9** | **Consensus*** |
| --- | --- | --- | --- | --- |
| **Microbiological Burden** | Pathogen-specific resistance rates | 7.0 (2.0) | 76% | Yes |
|  | Multidrug resistance rates | 7.0 (2.0) | 76% | Yes |
|  | Year-on-year resistance trends | 7.0 (2.0) | 76% | Yes |
| **Patient Impact** | Incidence of resistant infections | 8.0 (1.0) | 94% | Yes |
|  | Length of stay | 7.0 (2.0) | 71% | No |
|  | Time to effective therapy | 7.0 (2.0) | 65% | No |
| **Stewardship Systems** | Staff training and education | 8.0 (1.0) | 88% | Yes |
|  | Clinical Decision Support Systems | 7.0 (2.0) | 76% | Yes |
|  | Audit and feedback mechanisms | 7.0 (2.0) | 76% | Yes |
| **AMR Bioeconomics** | Incremental cost vs susceptible infections | 8.0 (1.0) | 94% | Yes |
|  | Attributable mortality cost | 8.0 (2.0) | 88% | Yes |
| **Prescribing Practices** | WHO AWaRe usage patterns | 7.0 (2.0) | 71% | No |

*Consensus defined as ≥75% agreement (ratings 7-9) with IQR ≤2

### **Supplementary Table S2: Consensus Evolution Across Rounds**

**Abbreviations:** WHO AWaRe = World Health Organization Access, Watch, Reserve; AMS = Antimicrobial Stewardship; MDR = Multi-Drug Resistant; ASI = Antimicrobial Spectrum Index

| **Indicator** | **Round 1 (n=17)** | **Round 2 (n=7)** | **Round 3 (n=7)** | **Consensus Trend** |
| --- | --- | --- | --- | --- |
| Incidence of resistant infections | 8.0 (1.0) | 7.0 (2.0) | 8.0 (0.0)* | Strengthened to unanimous |
| Pathogen-specific resistance rates | 7.0 (2.0) | 8.0 (2.0) | 7.0 (2.0) | Maintained strong |
| WHO AWaRe usage patterns | 7.0 (2.0) | 8.0 (2.0) | 8.0 (2.0) | Strengthened and maintained |
| Multidrug resistance rates | 7.0 (2.0) | 6.0 (3.0) | - | Lost in Round 3 |
| Attributable mortality cost | 8.0 (2.0) | 6.0 (2.0) | 7.0 (1.0) | Recovered consensus |
| AMS intervention cost-effectiveness | 6.0 (2.0) | 5.0 (2.0) | 7.0 (1.0) | Gained consensus |
| Audit and feedback mechanisms | 7.0 (2.0) | 7.0 (2.0) | - | Maintained through Round 2 |
| Antimicrobial Spectrum Index | 4.0 (2.0) | 5.0 (1.0) | - | Declined priority |

*Unanimous agreement (IQR = 0.0)

**Supplementary Table S3: step-by-step conversion of raw data to overall score**

| **Domain (Weight)** | Raw Data Inputs | Step 1 – Feature Extraction | Step 2 – Normalisation / Penalties | Step 3 – Domain Score (0–100) | Contribution to Overall Score |
| --- | --- | --- | --- | --- | --- |
| **Resistance (25%)** | MDRO episode count; total antibiotic episodes; specialty and monthly flags | Compute prevalence rate = mdro_episodes_count / total_episodes; derive specialty and monthly stratifications. | Scale prevalence: resistance_score = min(100, prevalence_rate * 1000). (Low prevalence yields higher scores.) | resistance_score | resistance_score×0.25 |
| **Effectiveness (25%)** | Prescription timestamps, administration timestamps, admission/discharge dates. | time_to_admin = admin_time - prescription_time; timeliness_rate = timely_admin / total_prescriptions; LOS per encounter. | timeliness_score = timeliness_rate * 100; los_score = max(0, 100 - (avg_los_days - 7) * 5). (Penalises LOS beyond 7 days.) | effectiveness_score=(timeliness_score×0.7)+(los_score×0.3) | effectiveness_score×0.25 |
| **Monitoring (20%)** | Culture linkage within ±1 day, AWaRe category counts, susceptibility confirmations. | culture_guided_rate = guided_prescriptions / total_prescriptions; tally Access/Watch/Reserve prescriptions; identify susceptibility-confirmed cases. | aware_compliance=total(Access×1.0)+(Watch×0.5)+(Reserve×0.1) ×100; dasc_score=totalsusceptibility_confirmed ×100. | monitoring_score=(culture_guided_rate×40)+(aware_compliance×30)+(dasc_score×30) | monitoring_score×0.20 |
| **Adoption (10%)** | Access-class prescriptions (compliance proxy) and counts per specialty per month. | guideline_compliance_rate=total_prescriptionsaccess_prescriptions ; compute prescription counts by specialty. | specialty_variation=mean(counts)std(counts) ; variation_score=max(0,100−specialty_variation×100). (Penalises variation.) | adoption_score=(guideline_compliance_rate×60)+(variation_score×40) | adoption_score×0.10 |
| **Processes (10%)** | Sequential antibiotic prescriptions within 7 days, drug spectrum labels. | Identify de-escalation events (broad → narrow) and compute de_escalation_rate=total_switchesevents ; calculate switch intervals. | switch_time_score=max(0,100−(avg_switch_time_hours−24)×2). (Penalises delays beyond 24h.) | processes_score=(de_escalation_rate×60)+(switch_time_score×40) | processes_score×0.10 |
| **Systems (10%)** | Infection-related LOS and readmission proxies. | Derive infection cohort; compute avg_los_infection and infection_readmission_rate=total infection cohortproportion with LOS>14 days . | los_score=max(0,100−(avg_los_infection−7)×5); readmission_score=max(0,100−infection_readmission_rate×100). | systems_score=(los_score×60)+(readmission_score×40) | systems_score×0.10 |
| **Overall** | All six domain scores. | — | — | overall_score=∑(domain_score×domain_weight) | Final REMAPS index reported on 0–100 scale. |
